# Methodological considerations in PISCES 3: a randomized, placebo-controlled study of intracerebral stem cells in subjects with disability following an ischemic stroke

**DOI:** 10.3389/fstro.2023.1182537

**Published:** 2023-07-04

**Authors:** Daniel T. Laskowitz, Keith W. Muir, Sean I. Savitz, Lawrence R. Wechsler, Julie G. Pilitsis, Scott Y. Rahimi, Richard L. Beckman, Vincent Holmes, Peng R. Chen, Laura Juel, Deborah Koltai, Brad J. Kolls

**Affiliations:** ^1^Duke University School of Medicine, Duke Clinical Research Institute, Durham, NC, United States; ^2^Department of Neurology, Duke University School of Medicine, Durham, NC, United States; ^3^Queen Elizabeth University Hospital, Institute of Neuroscience and Psychology, University of Glasgow, Glasgow, United Kingdom; ^4^Institute for Stroke and Cerebrovascular Disease, University of Texas Health Science Center, Houston, TX, United States; ^5^Department of Neurology, Perelman School of Medicine, University of Pennsylvania, Philadelphia, PA, United States; ^6^Department of Neuroscience and Experimental Therapeutics, Albany Medical College, Albany, NY, United States; ^7^Department of Neurosurgery, Medical College of Georgia, Augusta, GA, United States; ^8^ReNeuron Inc, Pencoed, United Kingdom; ^9^The Vivian L. Smith Department of Neurosurgery, University of Texas Health Science Center, Houston, TX, United States; ^10^Department of Physical Therapy and Occupational Therapy, Duke University School of Medicine, Durham, NC, United States; ^11^Department of Psychiatry, Duke University School of Medicine, Durham, NC, United States

**Keywords:** stroke therapy, ischemic stroke (IS), cell based intervention, stem cell, stroke outcome measures

## Abstract

**Background and hypothesis:**

At present, there are no medical interventions proven to improve functional recovery in patients with subacute stroke. We hypothesize that the intraparenchymal administration of CTX0E03, a conditionally immortalized neural stem cell line, linked with a standardized rehabilitation therapy regimen for the upper limb, would improve functional outcomes in patients 6–12 months after an index ischemic stroke.

**Study design:**

PISCES III was designed as a multicenter prospective, sham-controlled, outcome-blinded randomized clinical trial. Eligibility required a qualifying ischemic stroke 6–12 months prior to surgical intervention. Patients must be between 35 and 75 years of age and have residual moderate or moderately severe disability (mRS 3 or 4), with the preservation of some residual upper limb movement. All patients received a standardized regimen of home physical therapy following the intervention.

**Study outcomes:**

The primary outcome measure is improvement in the modified Rankin Scale (mRS) of disability at 6 months post treatment. Secondary outcomes include assessment of activities of daily living (Barthel Index), functional mobility (Timed Up and Go; Fugl Meyer Assessment), neurological impairment (NIHSS), upper limb function (Chedoke Arm and Hand Inventory), as well as patient related quality of life and global rating scales.

**Discussion:**

PISCES III was designed as a randomized trial directly comparing the effects of intraparenchymal injection of a conditional stem cell line vs. sham procedure in patients with subacute stroke. This is one of the first studies of this type to include a standardized minimum rehabilitation protocol. As there are a limited number of studies evaluating invasive stem cell administration in the chronic setting of CNS injury, study design considerations are discussed.

**Clinical Trial Registration:**

https://clinicaltrials.gov/, identifier NCT03629275.

## Introduction

Stroke remains a leading cause of morbidity in the Unites States, with an estimated 800,000 new strokes occurring annually (Virani et al., 2020; Tsao et al., 2022). Unlike traditional neuroprotective and reperfusion therapies, cell-based interventions may improve long term plasticity and recovery (Hassani et al., 2012). If successful, such therapies would allow for interventions in the subacute and chronic setting and offer the potential for reducing the burden of disability in a much larger population than is eligible for reperfusion. However, there are a number of unique challenges associated with the design of randomized trials of interventional cell therapy to improve the trajectory of motor limb recovery during the subacute and chronic stages of stroke, including optimization of dosing, route of administration, timing relative to stroke, appropriate sham controls, and functional endpoints that are sensitive to the therapeutic intervention.

The PISCES III trial (NCT03629275) was designed to demonstrate efficacy of stereotactic implantation of conditionally immortalized neural stem cells (CTX0E03) on functional outcomes in disabled stroke survivors. This study followed prior open-label clinical studies demonstrating that stereotactic intraparenchymal administration of CTX0E03 human neural stem cells was safe, feasible, and associated with improvement in upper limb movements (Kalladka et al., 2016; Muir et al., 2020). Although recovery of arm and hand movements may be associated with clinically meaningful gains in functional status and quality of life, traditional broad functional endpoints commonly used in acute reperfusion and neuroprotection trials, such as the modified Rankin score (mRS) emphasize ambulation and may be insufficiently sensitive to detect meaningful gains of function, especially in smaller clinical trials (Cramer et al., 2021). Thus, in addition to clinical efficacy, the PISCES III study was designed to identify clinically relevant measures of upper extremity function and quality of life.

## Methods and analysis

### Study design

**Figure 1:**
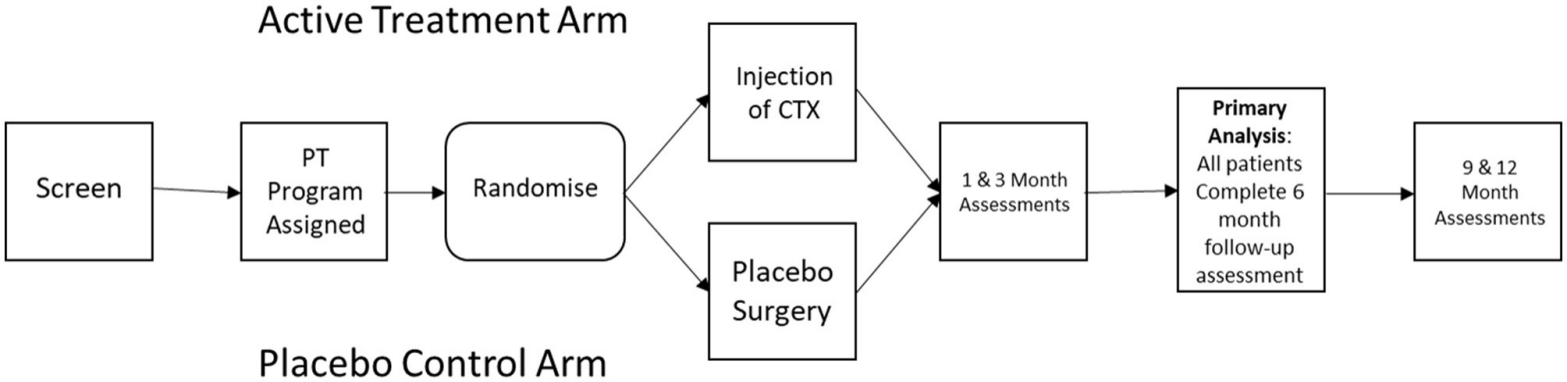
Schematic of study design.

As outlined in Figure 1, PISCES III was designed as a multicenter prospective sham controlled randomized clinical trial to evaluate efficacy of intraparenchymal injection of CTXE03, a conditionally immortalized neural stem cell line vs. sham procedure in patients with subacute stroke. Recruitment was facilitated by internet recruitment via a patient facing portal. A hub and spoke model was used, with 8 high-volume stereotactic surgical “hubs” and a larger number of assessment center “spokes”.

**Patient Population** (See trial synopsis in Appendix 1 for full inclusion and exclusion criteria).

#### Inclusion criteria

1. Written informed consent or witnessed informed consent in the event that the subject is unable to sign informed consent.
2. Ischemic stroke that includes the supratentorial region (including infratentorial stroke with supratentorial involvement) occurring within 6 to 12 months of the time that surgical intervention will be performed.
3. Age between 35 and 75 years of age (inclusive).
4. Qualifying stroke event confirmed by CT or MRI.
5. Must have current moderate or moderately severe disability as measured by modified Rankin Score of 3 or 4 due to the qualifying stroke event.
6. Must have some residual upper limb movement.
7. Must have sufficient cognitive and language abilities to comprehend verbal commands and to carry out the study assessments.
8. Sufficient putamen, globus pallidus, or caudate nucleus volume on the affected side to enable delivery of the CTX0E03.

#### Exclusion criteria

1. Modified Rankin Score of >1 prior to the Qualifying Stroke Event.
2. Stroke due to hemorrhage.
3. Neurosurgical pathway obstructed by vascular malformation or cavity.
4. Contraindication to CT or MRI imaging with contrast agents.
5. Inability to stop or transition off valproic acid or other demethylating agents or histone deacetylase inhibitors for 1 week before and 4 weeks following treatment.
6. Use of selective serotonin reuptake inhibitors (SSRI), unless the subject is on a stable dose that has been started at least 2-months before screening.
7. Inability to discontinue anticoagulation therapy for a required interval.
8. History of malignant disease within the last 5 years.
9. Clinically significant laboratory values that may impact the ability of the subject to safely participat in the entire study or any other conditions that would preclude safe participation.

### Randomization and blinding

Subjects were randomized for CTX0E03 injection or sham intervention at a 1:1 ratio following confirmation of subject eligibility and planned date of surgery. Block randomization was performed to avoid long runs of CTX0E03 DP or Placebo treatment. The randomization schedule and stratification scheme was pre-programmed into the electronic data capture system. The subject, investigator, and assessor were all blinded to treatment assignment.

### Cell-based intervention

CTX0E03 is a differentiated clinical-grade clonal human stem cell line derived from human cortical neuroepithelium. Cells were created from conditionally immortalized clonal neural stem cell line (Pollock et al., 2006) by incorporating a fusion protein comprising c-Myc and a modified estrogen receptor binding domain that is regulated by 4-hydroxytamoxifen (4-OHT). In the absence of 4-OHT, the cells undergo grow arrest and terminal differentiation (Pollock et al., 2006; Smith et al., 2012). Preclinical studies have demonstrated that administration of CTX0E03 cells are associated with improved functional outcomes in a variety of central and peripheral nervous system injuries, including a murine model of ischemia/reperfusion (Stroemer et al., 2009; Smith et al., 2012; Hicks et al., 2013), and peripheral nerve engraftment (O'Rourke et al., 2018). Although the exact mechanism(s) by which CTX0E03 cells improve functional recovery in remain incompletely characterized, this intervention is believed to exert its effects via paracrine mechanisms resulting in adaptive immunomodulatory and neurotrophic effects, and has also been demonstrated to promote neurogenesis and angiogenesis (Sinden et al., 2017; Stonesifer et al., 2017). It is notable that in preclinical models, a substantial proportion of cells remain viable and differentiate. For example, in a model of stroke, functional improvement was associated with survival of implanted CTX0E03 cells survived in both ischemic and contralateral tissue following middle cerebral artery occlusion (Stevanato et al., 2009). Notably, after intraparenchymal (but not intrathecal injection), there was significant graft survival, with dispersion from the injection track; moreover approximately 2% of CTX0E03 underwent neuronal differentiation and 20% underwent astrocytic differentiation (Pollock et al., 2006; Smith et al., 2012). Direct intraparenchymal implantation has proven safe in phase 1^4^ and phase 2^5^ clinical trials. Twenty million cells of CTX0E03 were administered in the treatment arm; this dosing was informed by these two prior studies in chronic stroke (Kalladka et al., 2016; Muir et al., 2020).

The mode of administration of CTX0E03 direct parenchymal injection. Administration of cell-based interventions following stroke can be accomplished via a variety of routes, including intravenous, intra-arterial, intrathecal, and intraparenchymal. Although each strategy has potential advantages and liabilities, at present there is no proven advantage from any specific mode of administration. For example, more than half of all clinical trials utilize the least invasive strategy of intravenous administration, which has the potential disadvantage of reduced penetration in the CNS compartment following filtration through the pulmonary vasculature Although the number of cells ultimately reaching the brain are a function of both cell type and timing from injury, in preclinical models it has been estimated that only approximately 1% of intravenously administered cells may reach the brain (Chen et al., 2001), Cell delivery into brain may increase significantly with intra-arterial administration (Rodriguez-Frutos et al., 2016), although direct carotid injection may raise concerns over microembolization. Given the prior safety studies, PISCES 3 utilized the more invasive direct intraparenchymal injection of cell-based interventions to optimize the number of cells at the site of injury.

### Primary outcome

The primary efficacy objective of the study was clinically relevant improvement in the modified Rankin Score (mRS) at 6 months post-intervention. The primary efficacy response was defined by the proportion of responders to non-responders. Response was defined as any decrease in mRS at 6 months relative to baseline. To assure reproducibility between sites, key assessments were to be recorded to allow for central adjudication procedures. The central reviewers will receive the mRS videos in cohorts, in a masked manner and without knowledge of the site nor the visit date associated with the mRS videos. Primary and secondary endpoints are included in Table 1.

**Table 1:** Primary and secondary clinical endpoints.

**Primary**	
Modified Rankin Score (mRS)	Global Assessment of Function (primary endpoint is proportion of patients with positive shift in mRS at 6 months)
**Secondary**	
Barthel Index (BI)	Activities of daily living
Chedoke Arm and Hand Inventory (CAHI)	Upper extremity function
National Institutes of Health Stroke Scale (NIHSS)	Neurological Deficit
Fugl-Meyer Assessment (FMA)	Stroke-specific index of sensorimotor recovery
Timed Up and Go (TUG)	Functional mobility; lower limb function
Health-related quality of life	Health-related quality of life
Abbreviated stroke impact scale (aSIS); Euroqual 5 Dimensions (EQ-5D-5L)	Patient reported outcome measure of health related quality of life
Subject Global Rating of Change	Patient reported outcome measure of global improvement
Symbol Digit Modalities Test (SDMT)	Cognition: executive sequencing speed
Lexical/Semantic Controlled Oral Word Association	Cognition: executive verbal fluency
Multilingual Naming test (MiNT)	Cognition: confrontation naming
Montreal Cognitive Assessment (MoCA)	Cognition: a measure of global cognition, including orientation, executive, memory, language, and visual-spatial skills.

### Secondary outcomes

The Barthel Index (Mahoney and Barthel, 1965) was included as a key secondary endpoint due to its ease of performance and clinical relevance as a measure of functional independence (McDowell and Newell, 1996). Importantly, neither the mRS nor the BI is optimized for upper extremity function, which was a key indicator of therapeutic efficacy based on prior studies (Muir et al., 2020). To address this issue, PISCES III incorporated the Chedoke Arm and Hand Inventory (CAHAI) (Barreca et al., 2004, 2005). The Timed up and go (TUG) was incorporated to provide a measure of functional mobility (van de Port et al., 2008; Faria et al., 2012). Additionally, the Fugl-Meyer Assessment (FMA) of sensorimotor recovery (Fugl-Meyer et al., 1975; Gladstone et al., 2002) and the National Institute of Health Stroke Scale (NIHSS) were included as measures of stroke-related neurologic deficits (Brott et al., 1989; Anemaet, 2002). Other secondary endpoints included patient reported outcome measures and quality of life assessments. Finally, serial neurocognitive assessment was performed by using a truncated battery of tests designed to minimize patient burden, while screening multiple cognitive domains.

### Sample size estimates

The appropriate powering of a subacute stroke trial is limited by our incomplete understanding of the natural history of post-stroke motor recovery beyond the first 3–6 months in different patient populations. Ultimately, the inclusion of non-treated patients that exhibit greater than expected natural recovery in the subacute setting would potentially jeopardize the power of a study to demonstrate improvement that is a function of intervention. This proportion of patients might be expected to be larger in the motivated, closely monitored patients receiving focused physical therapy. Thus, for purposes of powering PISCES III, the proportion of non-treated patients expected to spontaneously improve enough to meet the primary endpoint was set as 12.5% (as compared to overall response rate of 35% necessary to meet the primary endpoint). Assuming equal probabilities of shifting from a baseline mRS of 3 to 2 or 4 to 3, and equal baseline mRS 3:4 distribution, and assuming a placebo response rate of 12.5% and a treatment effect in the CTX0E03 group of 22.5%, it was determined that a total sample size of 110 subjects was needed for a minimum of 80% power at a 5% significance level.

### Statistical analysis

As noted, the primary efficacy endpoint was the change in modified Rankin Score (mRS) determined from centralized adjudication center at 6 months post implant procedure. Subjects with no measure of mRS at 6 months will be considered as non-responders. Logistic regression analysis will be used to test the null hypothesis that there is no effect of CTX0E03 on the odds of a response. Logistic regression was chosen for the primary analysis to permit adjustment for stratification factors. The required sample size for two-sided likelihood ratio test of an odds ratio statistic with matching sample size assumptions is identical to the sample size required for the approximate z-test (e.g., n=55 in each group). The randomization stratification factors will be added as covariates in the logistic model. Stratification will be based on time since stroke (6–9 m; >9m); and baseline stroke severity (mRS). The modified intention to treat (mITT) population of patients who were randomized and received general anesthetic will be used for the primary analysis. Secondary endpoints that are interval or ratio in nature, or potentially have a large number of ordinal categories will be examined using the non-parametric Wilcoxon rank-sum test.

## Discussion

Cell based restorative interventions such as CTX0E03 offer the promise of improving function and quality of life in patients suffering from chronic disability due to stroke. In the chronic setting, early clinical evidence suggests that intracerebral stereotactic cell-based interventions promote recovery by enhancing neurotrophic support, reducing inflammation and promoting neurogenesis and angiogenesis (Wechsler et al., 2018). The evaluation of cell-based intervention for patients with chronic stroke remains a dynamic and rapidly evolving area of translational and clinical research, and multiple cell based interventions are currently being evaluated in the setting of stroke, including administration of non-neural stem cells, such as umbilical cord blood (Laskowitz et al., 2018); allogeneic marrow-derived cells (Hess et al., 2017), and allogeneic modified marrow-derived mesenchymal cells (Steinberg et al., 2016). The mechanistic basis by which cell-based interventions improve outcomes following stroke remain incompletely defined. Although paracrine effects associated with release of trophic factors have been demonstrated to enhance neurogenesis, angiogenesis plasticity and survival, immunomodulation is believed to play an important role. It is notable that in preclinical models, a substantial proportion of cells remain viable and differentiate. For example, in a model of stroke, functional improvement was associated with survival of implanted CTX0E03 cells survived in both ischemic and contralateral tissue at 1 month following middle cerebral artery occlusion (Stevanato et al., 2009). Following intraparenchymal (but not intrathecal) administration, there was significant graft survival, with dispersion from the injection track; moreover approximately 2% of CTX0E03 cells underwent neuronal differentiation and 20% underwent astrocytic differentiation (Pollock et al., 2006; Smith et al., 2012). Based on promising findings in earlier stage clinical trials suggesting CTX0E03 improves upper extremity function (PISCES 1 and 2), PISCES-3 was designed as a phase IIB trial with a sham control. The sham procedure-controlled design and blinded outcome evaluation in PISCES-3 address concerns that previous studies of potential cell therapies in subacute and chronic stroke have the potential to over-estimate possible benefits through a combination of placebo effects and observation of natural recovery.

The choice of a primary endpoint that is clinically relevant and sensitive to the therapeutic intervention is critical to demonstrate efficacy of cell-based intervention trials in chronic stroke patients. Traditionally, clinical trials evaluating the efficacy of acute neuroprotectant and reperfusion studies have focused on intuitive global functional outcome measures such as 90-day assessment of modified Rankin score (mRS) (Quinn et al., 2007). There are a number of advantages associated with the mRS as a primary endpoint, including simplicity of use and clinical relevance. Inter-rater reliability of mRS may be improved by use of the Rankin Focused Assessment tool (Saver et al., 2010) and validated scripts for telephone interviews (Janssen et al., 2010). Indeed, the mRS has become the standard-bearer for acute stroke interventions, and was suggested by the FDA as the primary endpoint for PISCES III. The Barthel Index also represents a common assessment incorporated into stroke trials, and this was included as a key secondary endpoint due to its ease of performance and clinical relevance as a measure of functional independence, with focus on personal care and mobility (Mahoney and Barthel, 1965). However, the utility of the mRS has been called into question, as it is primarily weighted toward ambulation, and may not capture other domain specific differences in function (Erler et al., 2022).

Importantly, neither the mRS nor the Barthel Index is optimized for upper extremity function, which was believed to be key indicator of therapeutic efficacy based on prior studies (Muir et al., 2020). Thus, in addition to these global assessments commonly used in acute stroke trials, it is important to include clinically relevant domain specific assessments in subacute stroke studies (Cramer et al., 2021). To address this issue, earlier studies of parenchymal stem cell administration have employed the Action Research Arm Test (ARAT). Although there are a number of advantages to this assessment including minimal patient burden, disadvantages include the potential for floor and ceiling effects, and lack of sensitivity in patients with more severe impairment (van der Lee et al., 2002). To address these deficiencies, PISCES III incorporated an alternative measure of upper extremity function, the Chedoke Arm and Hand Inventory (CAHAI) (Barreca et al., 2004, 2005). This assessment includes 13 tasks that are graded on a 7-point scale. Although it is slightly longer than the ARAT and requires some degree of training, advantages include improved ecological validity, as real life tasks are incorporated, as well as its responsiveness to change over time (Barreca et al., 2005).

In addition to these assessments of global function and upper extremity function, PISCES III incorporated a number of exploratory assessments of sensorimotor function, patient reported outcome measures, and cognition. In general, these assessments were chosen based on translation of real-world function. For example, the Timed up and go (TUG) was incorporated to provide a measure of functional mobility (van de Port et al., 2008; Faria et al., 2012) rather than gait velocity (6 minute walk test, Butland et al., 1982), as the TUG has been shown to be predictive of an individual's ability to walk unaided in both an indoor and outdoor environment (van de Port et al., 2008). Additionally, the Fugle-Meyer Assessment (FMA) of sensorimotor recovery after a stroke was incorporated as a secondary endpoint (Fugl-Meyer et al., 1975; Gladstone et al., 2002). The FMA is a stroke-specific, performance-based impairment index designed to assess motor functioning, balance, sensation and joint functioning in subjects with post-stroke hemiplegia (Fugl-Meyer et al., 1975; Gladstone et al., 2002). Although less functionally relevant, the 15-item National Institute of Health Stroke Scale (NIHSS) was included as it is considered a prognostically important and sensitive measure of stroke-related neurologic deficits (Brott et al., 1989; Anemaet, 2002). Other important domains include patient reported health related quality of life (QoL), which was captured with the stroke-specific Stroke Impact Scale (Duncan et al., 2003), and general health related QoL scale, EQ5-D (Herdman et al., 2011). Subjects' perception of improvement or worsening in their condition was also collected using a global rating of change scale. Finally, serial neurocognitive assessment was performed by using a truncated battery of tests designed to minimize patient burden, while screening multiple cognitive domains. This battery included assessments of memory and orientation (Montreal Cognitive Assessment); linguistic confrontation naming (Multilingual Naming Test); executive verbal fluency (Lexical/Semantic Controlled Oral Word Association); and executive sequencing (Symbol Digit Modalities Test).

Concurrent rehabilitative therapies may play an important role in determining functional improvement following stroke, and the timing and extent of physical and occupational therapy should be carefully adjusted for. Although it is difficult to completely control for the effects of occupational therapy on outcome, as the extent and efficacy of rehabilitative efforts may be influenced by baseline disability, PISCES III addressed this issue by assigning a standardized upper extremity occupational therapy program (GRASP) to each subject prior to randomization (Harris et al., 2009), with the level of activity determined by the subject's baseline functional impairment score (Barreca et al., 2004, 2005). Rehabilitation therapy will be self-delivered by the subject, though monitored by phone once weekly by a therapist, and continued for 12 weeks following the surgical intervention. Compliance with the assigned physical therapy regimen was captured by having subjects complete an electronically recorded diary.

Another significant challenge associated with study design for cell-based intervention is defining the optimal timing of therapy relative to index stroke. Although the majority of spontaneous functional improvements may occur during the first 3–6 months after stroke (Jorgensen et al., 1995), the trajectory of recovery can be heterogeneous, and as noted above, may also be a function of concurrent rehabilitative efforts. Ideally, for the most unambiguous interpretation of therapeutic effect, the intervention should occur when serial neurological exams are stable, and the trajectory of natural recovery is minimal and predictable. For this reason, it has been proposed that study design for chronic stroke should include patients who are at least 6 months removed from the index stroke, and have demonstrated no change in deficit for at least 2 months (Savitz et al., 2014). At this time after stroke, cell therapy may provide a treatment option when no other proven therapies are available. This guidance was integrated into the initial study design of PISCES III, and in fact has been adopted for similar cell-based trials (Kondziolka et al., 2000; Steinberg et al., 2016).

In addition to the uncertainties defining the earliest treatment time point to minimize the effect of natural recovery, it is also important to define the latest post-stroke interval in which treatment would still be effective. Although it would seem intuitive that the potentiation of inherent plasticity by a cell-based intervention would decrease with increased time interval from stroke, this has never been definitively demonstrated. Adopting the most conservative strategy of defining a relatively short exclusionary time window for drug administration can jeopardize timely enrollment, which is often a challenge in subacute interventional stroke studies (Ferreira et al., 2019). Extrapolating the therapeutic window for cell-based intervention is difficult to predict from preclinical models, and future clinical studies should focus on defining whether therapeutic efficacy varies with chronicity of stroke.

### Ethics and dissemination

#### Ethical considerations

Safety and ethical considerations are important in the design of procedural studies (Miller and Kaptchuk, 2004) and play a particularly important role in the design of invasive cell-based interventions employing direct intraparenchymal delivery. Although every effort was made to minimize risk by selecting institutions and surgeons that had established a significant volume and safety history performing similar procedures, as well as standardizing the stereotactic procedures and post-operative monitoring (Olmsted et al., 2022), the intraparenchymal stereotactic injection of cells is, by nature, an invasive procedure with the potential complication rate of 1–2%, including hemorrhage (Muir et al., 2011). For this reason, we excluded patients with relatively mild deficits, and determined that the stroke qualifying event must leave the patient with a mRS of 3 or 4 (defining a patient who has lost functional independence and rendered dependent on others for activities of daily living). In this circumstance, meeting the primary endpoint of improving by at least one point on the mRS would allow functional independence for a patient with a pre-treatment mRS of 3, or allow independent ambulation or increased activities of daily living for a patient with a pretreatment mRS of 4. Although patients with a pre-treatment mRS of five were excluded as it was felt that they would be less likely to respond to therapy, in general, however patients with higher disability were more likely to express the largest improvement in wellbeing with a one point shift in mRS (Wang et al., 2020).

Another ethical issue inherent in the studies employing invasive procedure is the appropriate use of controls. Clearly, the maintenance of blinding is critical for the unbiased interpretation of the study data. For example, without a true contemporaneous control group, there is the potential for placebo effect from the performance of surgery (Albin, 2002; Redberg, 2014), anesthetic and accompanying medical intervention. Subjects who are aware that they have not received active treatment might potentially be less adherent to assessments and physiotherapy, and unmasking might bias the assessment of functional assessments by study personnel. Despite the importance of a control for maintaining study integrity, a true placebo control group would require exposing patients to the risks of craniotomy. Thus, to maintain blinding, in PISCES III, subjects randomized to the placebo treatment received the similar application of a stereotactic frame, and a burr hole was created that was similar to the active treatment group. However, to minimize potential risk, the burr hole was only partial skull thickness and the dura was never breached during this procedure. The burr hole and the scalp wound were closed in an identical fashion to the active treatment group with a low-profile metal plate that is fixed to adjacent bone and provides protection over the craniotomy site. The presence of a burr hole and scalp wound is noticeable by the subject and others (e.g., family and caregivers, hospital staff) and effectively masked the treatment received both for the subject and for clinical staff involved in the study. Subjects in both groups received identical general anesthetic protocols, post-surgical observation and monitoring, and discharge procedures. In this manner, with the exception of the surgeon and the operation room team, all study personnel involved in patient assessments remained blinded throughout the study. In the event of a potential post-surgical complication, an external unblinded study monitor was assigned to break the blind and assure appropriate treatment.

An additional concern in the evaluation of cell-based interventions in the potential for long term tumorigenicity. Initial development of CTX0E03 cell administration included single dose toxicology, which demonstrated no adverse effects in rats, mice and non-human primates. Although there was no evidence of long term tumorigenicity in preclinical models (including animals treated with tamoxifen, which might in theory reduce the differentiation of conditionally immortalized fusion protein), this was more difficult to assess, due to the lack of long term CTXE03 cell engraftment or survival. Of note, there was no evidence of tumorigenicity in the first two clinical stroke studies in which patients received the identical administration as in PISCES III intraparenchymal injection of CTXE03 at a dose of up to 20 million cells. Although the database was designated to be locked following the final 12-month assessment, a separate 14-year long term safety follow-up study is in development and was presented to all subjects.

#### Data monitoring body

A Data and Safety Monitoring Board (DSMB) was organized to provide safety oversight of the intervention through evaluation of clinical and safety data as deemed necessary. A statistician was available for preparing the data for review and for consultation, but was not designated as a voting member of the DSMB. The DSMB will be chaired by the unblended medical monitor. None of the DSMB members were investigators in this study. Details of all serious adverse events (SAEs) that occur in the study will be provided to members of the DSMB in time for each periodic review of the safety data. The DSMB also reviewed any new clinical or non-clinical safety information on the study subjects (CTX0E03 DP or Placebo) that became available during the trial and that could change the benefit risk ratio of the CTX0E03 DP.

## Conclusion

In summary, although a great deal of emphasis has been placed on acute neuroprotection and reperfusion strategies following stroke, a very significant pool of patients is left with chronic deficits that impair quality of life. Cell based interventions, such as the intraparenchymal administration of immortalized neuronal cell line CTX0E0 offer the possibility of improving functional recovery in patients with chronic deficits following stroke and have proven safe in pilot studies. However, a number of variables, such as the optimal route of delivery and timing of intervention relative to index stroke remain incompletely defined. The nature of endpoints and control groups, as well as the role of concurrent rehabilitative efforts must also be carefully considered in chronic stroke studies, and the degree of invasiveness carefully balanced against potential benefits. The PISCES III protocol was designed to address these challenges, and bring cell-based interventions closer to fulfilling the promise of reducing long-term disability from stroke.

## Data availability statement

The original contributions presented in the study are included in the article/Supplementary material, further inquiries can be directed to the corresponding author.

## Author contributions

SS, BK, SR, RB, VH, LW, DK, LJ, PC, and DL contributed to conception and design of the study. DL and BK wrote the first draft of the manuscript. All authors reviewed the manuscript and provide approval for publication of the content and contributed to manuscript revision, read, and approved the submitted version.
