# Supplementary material for: Methodological considerations in PISCES 3: a randomized, placebo-controlled study of intracerebral stem cells in subjects with disability following an ischemic stroke
Source: Front Stroke. 2023 Jul 4;2:1182537. doi: 10.3389/fstro.2023.1182537 (PMC12802619; doi:10.3389/fstro.2023.1182537)
Supplement: Supplementary file 1 [file Data_Sheet_1.pdf]

## PROTOCOL SUMMARY

| | |
|---|---|
| **Protocol Number:** | RN01-CP-0003 |
| **Title:** | A Randomized, Placebo-Controlled Study of the Efficacy and Safety of Intracerebral Stem Cells (CTX0E03 DP) in subjects with Disability Following an Ischemic Stroke (PISCES III) |
| **Phase:** | IIB |
| **Sponsor:** | ReNeuron Ltd |
| **Indication:** | Disability due to supratentorial ischemic stroke |
| **Investigational Product / Dose:** | CTX0E03 Drug Product (DP) / 20 million cells and excipient |
| **Route of Administration:** | Intracerebral implantation |
| **Comparator Drug:** | Not applicable |
| **Study Design:** | A randomized, placebo-controlled, multicenter study.<br><br>Subjects will be directed to self-refer to the study by accessing the study website (*www.PISCES3.org*). Following confirmation of eligibility criteria, subjects will be randomized in 1:1 ratio to receive either a single administration of CTX0E03 DP by intracerebral implantation or receive placebo surgery only. Subjects will be followed for 12 months after CTX0E03 DP administration or placebo surgery with follow-up assessments occurring at Day 1, 7, and Months 1, 3, 6, 9, and 12.<br><br>All eligible subjects will be assigned a standardized Physical Therapy (PT) program (i.e. GRASP) based on their baseline functional impairment (CAHAI). Subjects will be expected to complete their PT exercises daily, and independently or with a family member/carergiver if required for 12 weeks post-surgery.<br><br>A separate 14-year long term safety follow-up study is in development which will be presented to all subjects enrolled within the RN01-CP-0003 (PISCES III) study for their inclusion once the study has started. |
| **Study Duration:** | Enrollment period: 11 months (estimated)<br>Total study length: 29 months (estimated) |
| **Number of Subjects/Sites:** | Approximately 110 subjects will be enrolled from approximately 30 Hub and Spoke sites in the USA and EU |
| **Primary Objective:** | To assess the efficacy of intracerebral CTX0E03 DP by change in degree of dependency and disability as measured by modified Rankin Score (mRS). |
| **Secondary Objectives:** | • To assess the effect of CTX0E03 DP treatment on change in:<br>o Independence in completing activities of daily living (ADL)<br>o Upper limb functional performance<br>o Functional Mobility<br>o Cognitive function<br>o Neurological impairment<br>o A stroke-specific impairment index |

| | |
|---|---|
| | • To measure the Subjects' global rating of change<br>• To measure quality of life<br>• To assess the safety and tolerability of intracerebral CTX0E03 DP:<br>  ○ Measured by adverse events reports and medical device adverse events/incidents reports<br>  ○ Measured by biochemistry and hematology laboratory testing<br>• Monitoring for development of anti-HLA antibodies |
| **Subject Population:** | Subjects will be in the stable chronic stage of stroke (6 to 12 months post stroke) with moderate or moderately severe disability as a result of an ischemic stroke. |
| **Inclusion Criteria:** | 1. Written informed consent or witnessed informed consent in the event that the subject is unable to sign informed consent due to paresis of the affected arm.<br>2. Ischemic stroke that includes the supratentorial region (including infratentorial stroke with supratentorial involvement) occurring within 6 to 12 months of the time that surgical intervention will be performed (Qualifying Stroke Event)<br>3. Aged between 35 and 75 (inclusive)<br>4. Qualifying Stroke Event must be confirmed by CT or MRI<br>5. Must have current Moderate or Moderately Severe disability as measured by modified Rankin Score = 3 or 4 due to the Qualifying Stroke Event<br>6. Must have some residual upper limb movement as defined by the GRASP manual e.g. ability to actively shoulder shrug against gravity and wrist extension (palpable by the investigator; or visible lift of the fingers with hand resting on a table)<br>7. Must have sufficient cognitive and language abilities to comprehend verbal commands and to carry out the study assessments<br>8. No medical conditions that would preclude neurosurgery with appropriate preparation and management.<br>9. Sufficient putamen, globus pallidus or caudate nucleus volume on the affected side to enable delivery of the CTX0E03 DP<br>10. Able to attend study related Visits and complete any diary, telephone or questionnaire assessments<br>11. Females of childbearing potential (FOCBP), (or within 2 years of last menstrual cycle) must have a confirmed negative pregnancy test at time of treatment and agree to use two reliable methods of contraception (e.g. oral contraceptive and condom, intra-uterine device (IUD) and condom, diaphragm with spermicide and condom) for six months following surgery<br>12. Sexually active males with partners who are FOCBP must be willing to use a reliable method of contraception (e.g. barrier and spermicide or as described above) for six months following surgery. |
| **Exclusion Criteria:** | 1. Permanent disability corresponding to a Modified Rankin Score of >1 prior to the Qualifying Stroke Event.<br>2. Stroke due to hemorrhage (whether caused by atherosclerotic disease or due to atypical cause), or stroke known or suspected of being caused-by, or related-to, connective tissue disorder, congenital disorder of the cerebral vessels or a disorder of thrombosis (See *Appendix 1*). Subjects with atrial fibrillation as a suspected cause of stroke are NOT excluded.<br>3. Neurosurgical pathway obstructed by vascular malformation or cavity<br>4. History of neurological or other disease resulting in significant functional impairment (e.g. Parkinson's disease, motor neuron disease, moderate dementia, arthritis, contractures or fixed anatomical abnormality).<br>5. Any contraindications to either CT scan or MRI e.g. presence of a magnetic-sensitive cardiac pacemaker, metal fragments in eye, contrast allergy etc.<br>6. Inability to stop or transition off valproic acid or other demethylating agents |

| | |
|---|---|
| | or HDAC inhibitors for 1 week before and 4 weeks after treatment with CTX0E03 DP<br>7. Use of selective serotonin reuptake inhibitors (SSRI), unless the subject is on a stable dose that has been started at least 2-months before screening (V1)<br>8. Use of antispasticity medications (excluding oral antispasticity medications if they have been taken regularly for at least four months prior to treatment with CTX0E03 DP)<br>9. Inability to discontinue anticoagulation therapy for a required interval (see Section *7.2*).<br>10. Subjects with a severe comorbid disorder that has reasonable likelihood of limiting survival to less than 24 months.<br>11. History of malignant disease within the last 5 years, (excluding benign tumours such as non-melanoma skin cancer, cervical carcinoma in situ, superficial bladder cancer, benign polyps).<br>12. Any history of primary or secondary brain malignant disease.<br>13. Subjects who have previously participated in a cell-based therapy study at any time or in any other study involving an investigational product or rehabilitation study within the last 30 days<br>14. Any clinically significant laboratory values, including positive Class I HLA antibodies specific for CTX0E03, during screening (refer to Section *6.2.2.1* for more details on HLA antibodies<br>15. Inability to adhere to the study post-surgery upper limb standard Physical Therapy regimen e.g. excessive spasticity or pain<br>16. Planned initiation of any other new physical therapy regimen within 6-months post-treatment<br>17. Any other conditions that, in the opinion of the investigators, would preclude safe and/or effective participation |
| **Description of Treatment:** | Subjects randomized to active treatment will receive an intracerebral implantation, (directed to the putamen, globus pallidus or caudate nucleus on the affected side), of 20 million CTX0E03 DP cells in 400 µL suspension. This is delivered using the Sponsor`s Implantation Set during a single neurosurgical procedure using a stereotactic frame or similar device, and administered as an intracerebral implantation through one or more burr holes.<br><br>Subjects randomized to the Placebo treatment will also have a stereotactic frame or similar device applied and a burr hole created identical to the active treatment group. The burr hole will be partial thickness and the dura will not be breached during this procedure. The burr hole and scalp wound will be closed with a burr hole cover identical to the active treatment group Subjects.<br><br>All subjects will receive a general anesthetic and will be kept under observation for a minimum of 4-6 hours after the completion of surgery. Discharge will be as per hospital standard of care. The next Visit assessment is scheduled the day following surgery. |
| **Subject Duration:** | All subjects will be followed for a total of 12 months post implantation of CTX0E03 DP or placebo surgery.<br><br>A separate 14-year long term safety follow-up study is in development which will be presented to all subjects within the RN01-CP-0003 (PISCES III) study for their inclusion once the study has started. |
| **Subject Visits / Assessments:** | All subjects will attend a total of 11 study visits whilst participating in the study. Visits 1, 2, 6-9) will occur at the Spoke site; Visits 3, 4 and 5 will occur at the Hub site. |

| | |
|---|---|
| **Monitoring of Safety and Tolerability:** | Study safety oversight will be monitored by a DSMB.<br><br>Safety monitoring will include a combination of Adverse Event reporting, clinical evaluations and safety laboratory evaluations. |
| **Assessment of Primary and Secondary Endpoints** | All Assessors will be trained and certified in the methodology of the primary and secondary efficacy endpoints. Additionally, the primary assessment of mRS will be video-recorded at baseline, Months 3, 6 and 12 and independently scored by a central reviewing committee. |
| **Additional Assessments:** | • Physical Therapy: A standardized Physical Therapy (PT) program (i.e. GRASP) will be assigned to each subject prior to randomization; the level of activity will be determined by the subject's baseline functional impairment score (CAHAI). The PT will be self-delivered by the subject and continue for 12 weeks post-surgery. Subjects will complete a diary of their PT. No new therapy programming should be planned until 6 months post-surgery.<br><br>• Evaluating the Effects of Masking: Subjects, Investigators, and Assessors will be asked a multiple choice question to describe which treatment allocation they/the subject is thought to have received.<br><br>• Evaluation of Usability of Medical Device used for Implantation of CTX0E03 DP. |
| **Efficacy Variables:** | • Primary<br>o Modified Rankin Scale (mRS)<br>• Secondary<br>o Barthel Index (BI)<br>o Timed Up and Go Test (TUG)<br>o Chedoke Arm and Hand Activity Inventory (CAHAI)<br>o Stroke-suitable cognitive battery test<br>o National Institute of Health Stroke Scale (NIHSS) Assessment<br>o Fugl-Meyer Assessment (FMA)<br>o Health related quality of life measures<br>o Assessment of Global Rating of Change<br>o Assessment of treatment allocation |
| **Safety Variables:** | • Pregnancy test (FOCBP or within 2 years of last menstrual cycle)<br>• General physical examination<br>• Vital signs (Heart Rate, Blood Pressure, Temperature, ECG, urinalysis)<br>• Basic hematology and biochemistry<br>• Liver function and Coagulation<br>• Brain imaging (MRI)<br>• Adverse Events<br>• Medical device adverse events/incident reporting<br>• HLA antibody assessment<br>• Completion of PT diary/e-diary |
| **Statistical Methods:** | Subjects will be randomized (1:1) to active CTX0E03 DP or Placebo. Stratification will be performed by: time since stroke (6-9m; >9m); and baseline stroke severity (mRS, 3; 4).<br><br>The primary efficacy objective of the study is to assess the effect of intracerebral CTX0E03 DP on the change in degree of dependency and disability from baseline as measured by the modified Rankin Score (mRS) at 6 months. The primary efficacy response will be dichotomous: a positive response to treatment (i.e. a |

| | |
|---|---|
| | decrease in mRS designates a "responder") at 6 months or no positive response.<br><br>It is assumed that approximately 12.5% of Placebo treated subjects and 35% of CTX0E03 DP treated subjects will show improvement. The study will be powered at 80%. |
